# d-Galactose-induced oxidative stress and mitochondrial dysfunction in the cochlear basilar membrane: an in vitro aging model

**DOI:** 10.1007/s10522-020-09859-x

**Published:** 2020-02-05

**Authors:** Bin Guo, Qing Guo, Zhan Wang, Jian-Bo Shao, Ke Liu, Zheng-De Du, Shu-Sheng Gong

**Affiliations:** 1 Department of Otorhinolaryngology, Beijing Friendship Hospital, Capital Medical University, 95 Yongan Road, Xicheng District, Beijing, 100050 China; 2 Department of Otorhinolaryngology, Qinghai University Affiliated Hospital, 29 Tongren Road, Qinghai, 810001 China; 3 Department of Otorhinolaryngology, Yidu Central Hospital, 4138 Linglong Shan South Road, Qingzhou, 262500 China; 4 Department of Biomedical Engineering, Tsinghua University, Shuangqing Road, Haidian District, Beijing, 100084 China; 5 Department of Otorhinolaryngology, Beijing Children’s Hospital, Capital Medical University, 56 Nanlishi Road, Xicheng District, Beijing, 100045 China

**Keywords:** d-galactose, Aging model, Presbycusis, Cochlear basilar membrane, Mitochondrial oxidative damage, Apoptosis

## Abstract

The cochlear basilar membrane (CBM) contains inner hair cells and outer hair cells that convert sound waves into electrical signals and transmit them to the central auditory system. Cochlear aging, the primary reason of age-related hearing loss, can reduce the signal transmission capacity. There is no ideal in vitro aging model of the CBM. In this study, we cultured the CBM, which was dissected from the cochlea of the C57BL/6 mice 5 days after birth, in a medium containing 20 mg/mL, 40 mg/mL, or 60 mg/mL d-galactose (d-gal). Compared with the control group, the levels of senescence-associated β-galactosidase were increased in a concentration-dependent manner in the CBM of the d-gal groups. In addition, levels of the mitochondrial superoxide and patterns of an age-related mitochondrial DNA3860-bp deletion were significantly increased. The ATP levels and the membrane potential of the mitochondrial were significantly decreased in the CBM of the D-gal groups compared with the control group. Furthermore, in comparison with the control group, damaged hair cell stereocilia and a loss of inner hair cell ribbon synapses were observed in the CBM of the d-gal groups. A loss of hair cells and activation of caspase-3-mediated outer hair cell apoptosis were also observed in the CBM of the high-dose d-gal group. These insults induced by D-gal in the CBM in vitro were similar to the ones that occur in cochlear natural aging in vivo. Thus, we believe that this is a successful in vitro aging model using cultured CBM. These results demonstrate the effects of mitochondrial oxidative damage on presbycusis and provide a reliable aging model to study the mechanisms of presbycusis in vitro.

## Introduction

Age-related hearing loss, also known as presbycusis, is a phenomenon related to aging in the peripheral auditory system and the central auditory system. About 80% of the elderly people suffer from a loss of hearing and consequential social impairment (Gates and Mills 2005). The basilar membrane in the cochlea of the peripheral auditory system contains critical sensorineural cells, inner hair cells (IHCs), and outer hair cells (OHCs), which convert sound waves into electrical signals and transmit them to the central auditory system (Fischer et al. 2019). The loss of critical sensorineural cells (Someya et al. 2007) or neural connections on the cochlear basilar membrane (CBM) is an important mechanism of presbycusis in mammals. When the peripheral auditory system undergoes aging in vivo, structural changes occur on the CBM, including loss of hair cells (Ohlemiller et al. 2010), loss of ribbon synapses between hair cells and spiral ganglion cells (Kujawa and Liberman 2015; Sergeyenko et al. 2013), and degeneration of stereocilia (Sekerková et al. 2011). However, no ideal aging model has been established on the CBM in vitro.

The Harman's free radical theory points out that increased oxidative stress and an accumulation of mutations of the mitochondrial DNA are key causes of aging (Harman 2006). Aging is associated with mitochondrial dysfunction due to the accumulated mutations of the mitochondrial DNA induced by oxidative stress (Conley et al. 2007). Previous animal models have provided reliable research platforms for studying aging mechanisms and age-related diseases. Researchers have proposed a method to accelerate the aging of rodents by using d-galactose (d-gal). This is gradually being recognized as a valid rodent model to study aging mechanisms (Haider et al. 2015; Ho et al. 2003).

d-Gal is a reducing sugar and is metabolized into glucose at normal concentrations. When concentrations reach higher than the ideal values, it is oxidized by galactose oxidase to form hydrogen peroxide, which can react with a reduced form of iron to form hydroxide ions; also converted into aldose through the action of galactose oxidase. In addition, d-gal reacts with amines to form an unstable compound. This compound can react non-enzymatically with free amines of amino acids in proteins to form advanced glycation end products (AGEs). AGEs bind with their receptors, resulting in the formation of oxygen-derived free radicals and reactive oxygen species (ROS) (Parameshwaran et al. 2010; Tian et al. 2005).

Related experimental evidence has shown that d-gal can induce oxidative stress and mitochondrial damage in different tissues that cause symptoms similar to aging, or mimic the natural aging process in rats, mice, and drosophila (Cui et al. 2004, 2006; Song et al. 1999; Wu et al. 2014). Chronic administration of d-gal for 6–8 weeks can induce aging in the cochlea of rodents in vivo (Du et al. 2012; Kong et al. 2006). However, these d-gal-induced aging models require a long time to develop and the aging process in these models can not easily controlled. Previous studies have established rapid aging models in the cochlear strial marginal cells (Zhao et al. 2013) and in the cortical astrocytes (Shen et al. 2014) in vitro using d-gal. However, whether exposure to d-gal in vitro can induce CBM senescence has not been documented. Therefore, we aimed at establishing an ideal and a reliable model of peripheral auditory aging in cultured CBM in vitro by exploring the mechanisms involved in presbycusis using d-gal-induced aging CBM.

## Experimental procedures

### Animals

The C57BL/6 mice 5 days after birth which were purchased from the Experimental Animal Center of the Capital Medical University. Animal care and experimental procedures were performed in accordance with the revised 1996 USA National Institutes of Health guidelines (NIH Publications). Animal experiments were approved by the animal research committee of Capital Medical University.

### CBM primary culture and senescence model induced by d-gal

Mice were euthanized with carbon dioxide and disinfected by immersion in 75% ethanol. The head was then removed, and the brain tissue was excised. Next, under a stereo dissection microscope, the osseous structure of the cochlea was acquired and the CBM quickly dissected. We separated the lateral wall of the stria vascularis and removed the tectorial membrane in Hank's Balanced Salt Solution (Life Technologies, Grand Island, NY, USA). Finally, the CBM was transferred into Dulbecco’s modified Eagles medium (DMEM)/F12 medium (Sigma-Aldrich, St. Louis, MO, USA) containing 10% bovine serum albumin (Sigma-Aldrich) and placed on the bottom of a culture dish for 24 h in a humidified incubator at 37 °C in 5% CO_2_ and 95% air. After 24 h, fresh DMEM/F12 medium was added with different concentrations of d-gal (Sigma-Aldrich) and the culture continued for another 48 h. The cultured CBMs were divided into four groups of 6 males, depending on the concentration of d-gal-control group: DMEM/F12 medium with 0 mg/mL d-gal; low-dose d-gal (L-d-gal) group: DMEM/F12 medium with 20 mg/mL d-gal; middle-dose d-gal (M-d-gal) group: DMEM/F12 medium with 40 mg/mL d-gal; and high-dose d-gal (H-d-gal) group: DMEM/F12 medium with 60 mg/mL d-gal.

### Senescence-associated β-galactosidase (SA-β-Gal) activity staining

The cultured CBM senescence assay was performed by staining for SA-β-Gal activity, and the pH of the SA-β-Gal staining solution was 6, following the manufacturer’s instructions and the steps outlined in previous reports (Benkafadar et al. 2019; Zhao et al. 2013). Briefly, the cell culture medium was removed and the CBM washed once with phosphate-buffered saline (PBS). Cultured CBMs were fixed for 15 min at room temperature and then incubated at 37 °C overnight, according to the senescence β-galactosidase kit protocol (Beyotime Biotechnology, Shanghai, China). Development of a blue color indicated SA-β-Gal activity. The stained image was observed using a light microscope and captured with a camera (Nikon, Tokyo, Japan) immediately after the procedure.

### Mitochondrial superoxide detection

Mitochondrial ROS levels of the CBM were detected using the mitochondrial superoxide indicator, MitoSOX (Invitrogen, Carlsbad, CA, USA). Briefly, the red mitochondrial superoxide indicator was dissolved following the manufacturer’s protocol. We mixed 1 mL medium with 1 uL of dissolved red mitochondrial superoxide indicator, and added them to culture dishes containing the CBMs. These steps were performed under the same parameters and culture conditions. The labeling reaction was performed at 37 °C for 15 min in a humified chamber. Nuclei were counterstained with 4′,6-diamidino-2-phenylindole for 5 min at room temperature. After washing once with PBS, the specimens were immediately observed under a fluorescence microscope (Leica DM2500, Wetzlar, Germany). The exposure intensity of these specimens was consistent. After exposure, these images were subjected to quantitative fluorescence analysis.

## Real-time polymerase chain reaction (RT-PCR)

The SYBR green RT-PCR assay was used to evaluate the relative quantity of mitochondrial DNA with a 3860-bp deletion. This common deletion (CD) increases with aging in the auditory system of mice (Du et al. 2019a), as reported previously (Zhang et al. 2009, 2013). Briefly, total DNA was extracted using a Genomic DNA Isolation kit (Tiangen Biotech, Beijing, China) and the DNA concentration of each specimen was measured (GeneQuant II, RNA/DNA calculator, GE Healthcare, Parramatta, Australia). Because mitochondrial 12S rRNA is rarely deleted, it can be used to represent the conserved segment. The forward and reverse primer sequences of 12S rRNA and the mitochondrial DNA 3860-bp deletion were defined in previous studies (Zhang et al. 2009, 2013) as: 12SrRNA forward, ACCGCGGTCATACGATTAAC; 12S rRNA reverse, CCCAGTTTGGGTCTTAGCTG; mitochondrial DNA 3860-bp deletion forward, TCATTCTAGCCTCGTACCAACA; mitochondrial DNA 3860-bp deletion reverse, GAGGTCTGGGTCATTTTCGTTA. The SYBR green RT-PCR assay was performed on a StepOne Real-Time PCR System (Applied Biosystems, Foster City, CA, USA). The PCR products of the mitochondrial DNA 3860-bp deletion were cloned and sequenced by an ABI Prism 377XL (Applied Biosystems). Relative expression of the mitochondrial DNA 3860-bp deletion was calculated by the 2^−ΔΔCt^ method.

### Measuring ATP levels and assessing mitochondrial membrane potential with JC-1

The luciferin-luciferase system was employed to quantify the ATP levels in these specimens according to the manufacturer’s instructions (Jiancheng Bioengineering Institute, Nanjing, China). A microplate reader was used to assess the relative ATP content of each specimen by bioluminescent intensity. In order to measure the mitochondrial membrane potential (MMP), a Mitochondria Isolation Kit (Beyotime Biotechnology, Haimen, China) was used to obtain mitochondria from each specimen. Then, the JC-1 probe (Jiancheng Bioengineering Institute) was employed to assess changes in the MMP according to the manufacturer's instructions. MMP kit with JC-1 is an ideal fluorescent probe for detecting mitochondrial membrane potential. JC-1 can detect cellular-, tissue-, or purified- MMP. And, JC-1 aggregates into the matrix of the mitochondria to form polymers. When detecting JC-1 monomer, the excited light can be set to 490 nm and the emission light can be set to 530 nm. When detecting JC-1 polymer, the excited light can be set to 525 nm and the emission light can be set to 590 nm. These steps were performed under the same parameters and culture conditions.

### Immunofluorescence staining

After d-gal treatment, cultured CBM were fixed with 4% paraformaldehyde for 3 min, and then rinsed with PBS. Specimens were subsequently blocked with 10% goat serum in 0.3% Triton X-100 for 30 min at room temperature. After blocking, we incubated the specimens at 37 °C overnight with the following primary antibodies diluted in PBS: monoclonal mouse anti-carboxyl-terminal binding protein 2 (CtBP2) (diluted 1:300; BD Biosciences, Franklin Lakes, NJ, USA) and polyclonal rabbit anti-myosin VIIa (diluted 1:400; Abcam, Cambridge, MA, USA). On the second day, CBMs were washed twice with PBS for 10 min each, and then incubated with the appropriate secondary antibodies and Alexa Fluor 488 Phalloidin (1:300; Invitrogen) at room temperature for 2 h. After a final wash in PBS for 10 min, a Leica TCS SP8 laser confocal scanning microscope was used for observing the specimens. Myosin VIIa labels hair cells, CtBP2 labels ribbon synapses, and Phalloidin labels hair cell stereocilia.

### Cochlear ribbon synapses and hair cell quantification

To quantify ribbon synapses, the total number of CtBP2-stained spots (red) was divided by the total number of IHCs. The well-characterized hair cell marker, myosin VIIa, was used to identify hair cells. Hair cell counting was performed as described previously (Wang et al. 2003). To quantify hair cell loss after d-gal treatments, we calculated the percentages of IHCs and OHCs that survived in three or four regions at the same magnification from each cultured CBM. The average survival percentage of hair cells was considered as a single specimen.

### Western blot analysis

Levels of cleaved caspase-3 (C-Cas3) protein in the CBM were determined by western blot analysis following the manufacturer's protocol. Briefly, we extracted total protein using radioimmunoprecipitation assay lysis buffer (Beyotime Biotechnology). An enhanced BCA Protein Assay Kit (Beyotime Biotechnology) was used to determine protein concentrations. Protein lysates were separated by sodium dodecyl sulfate-polyacrylamide gel electrophoresis, and then transferred onto polyvinylidene difluoride membranes. The membranes were incubated for 1 h in a blocking solution with 5% fat-free milk, then washed in Tris-buffered saline and incubated with the anti-C-Cas3 antibody (1:1000 dilution; Cell Signaling Technology, Beverly, MA, USA) at 4 °C overnight. After repeated washing, the membranes were treated with the appropriate horseradish peroxidase-conjugated secondary antibody (1:5000 dilution; Santa Cruz Biotechnology, Santa Cruz, CA, USA) for 1 h at room temperature. Finally, the membranes were visualized using BeyoECL Plus (Beyotime Biotechnology). Quantitative analysis of the detected bands was performed using Image-Pro Plus 6.0 software (Media Cybernetics, Rockville, MD, USA). β-Actin was used as an internal control for total protein.

### In situ apoptosis staining

Hair cell apoptosis in the CBMs was detected using a terminal deoxynucleotidyl transferase-mediated deoxyuridine triphosphate nick-end labeling (TUNEL) staining kit (TUNEL POD; Roche Molecular Biochemicals, Mannheim, Germany). After treatment with 10% goat serum in 0.1% TritonX-100 for 3 min, the labeling reaction, which contained terminal deoxynucleotidyl transferase, was performed for 1 h at 37 °C in a humidified chamber. The polyclonal rabbit anti-myosin VIIa antibody (diluted 1:400) was used to label the hair cells and a 4′,6-diamidino-2-phenylindole staining solution (Beyotime) was used to counterstain the nuclei. The CBMs were examined by laser confocal scanning microscopy (Leica TCS SP8).

### Statistical analyses

Data have been expressed as means  ± standard deviation. Statistical analyses were performed with SPSS 13.0 software (IBM, Chicago, IL, USA). A one-way analysis of variance was performed to compare groups. Differences between groups with *P* < 0.05 were considered statistically significant.

## Results

### Primary culture of CBMs

**Fig. 1:**
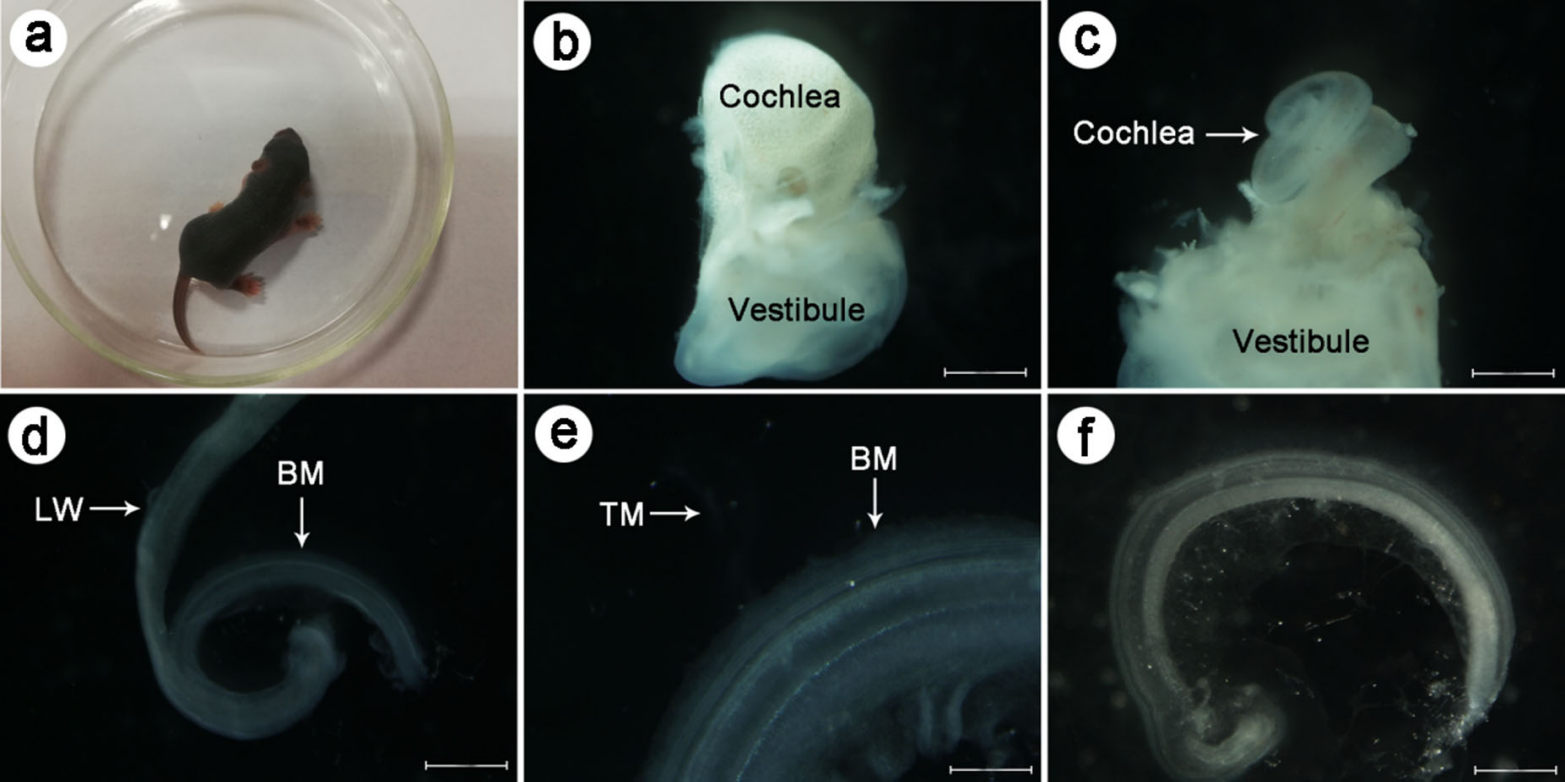
The process of cochlear basilar membrane primary culture. **a** The C57BL/6J mice 5 days after birth. **b** Complete cochlea and vestibule with bone labyrinth, which has been dissected out of the temporal bone under a stereo dissection microscope. **c** Full explanted cochlea that was dissected out of the bone labyrinth under a stereo dissection microscope. **d** Lateral wall (LW) of the stria vascularis is separated from the basilar membrane (BM) in Hank's Balanced Salt Solution. **e** The tectorial membrane (TM) is removed completely from the BM. **f** Cochlear basilar membrane cultured for 24 h in DMEM/F12 medium. Scale bar = 100 μm

Figure 1a–e shows CBMs that were separated from the cochlea of the C57BL/6J mice 5 days after birth. After a 24 h culture in DMEM/F12 medium, the CBMs grew close to the culture dish and numerous cells grew out of the CBM (Fig. 1f).

### Increased SA-β-gal in CBMs induced by d-gal

**Fig. 2:**
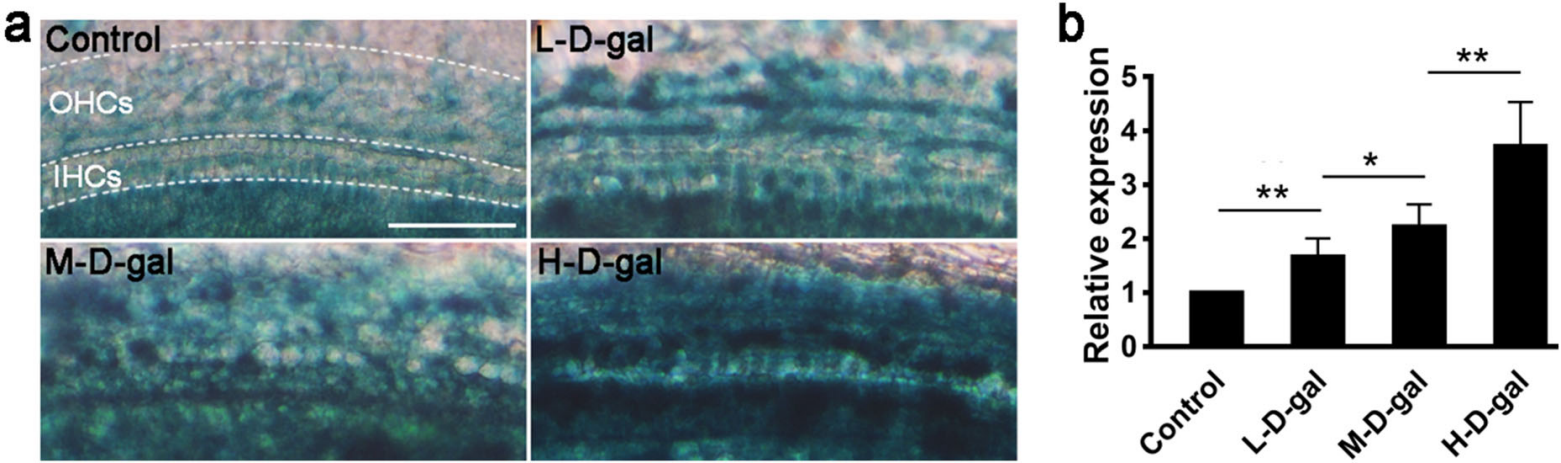
Senescence-associated β-galactosidase (SA-β-Gal) staining and relative expression in the control, low-dose d-gal (L-d-gal), medium-dose d-gal (M-d-gal), and high-dose d-gal (H-d-gal) groups. **a** Representative images of SA-β-Gal staining (blue) in the different groups. **b** Relative expression of SA-β-Gal in the different groups. Data are shown as means ± SD (n = 6 in each group). *P < 0.05, **P < 0.01. Scale bar = 50 μm. *OHCs* outer hair cells; *IHCs* inner hair cells.

To confirm that senescence was induced by d-gal in CBMs, SA-β-Gal staining was observed in the control group and the d-gal-treated group. As shown in Fig. 2a, SA-β-Gal blue staining in the d-gal-treated groups was significantly higher than in the control group. The level of SA-β-Gal staining was concentration-dependent with the H-d-gal group showing the highest number of SA-β-Gal-stained cells. Compared with the control group, the relative expression of SA-β-Gal in the L-d-gald-gal, M-d-gal, and H-d-gal groups were found to be increased by 1.67 ± 0.14-fold, 2.23 ± 0.17-fold, and 3.73 ± 0.33-fold, respectively (Fig. 2b). These results show that increasing concentrations of d-gal induced a corresponding degree of senescence in the CBM in vitro.

### Increased mitochondrial ROS in CBMs induced by d-gal

**Fig. 3:**
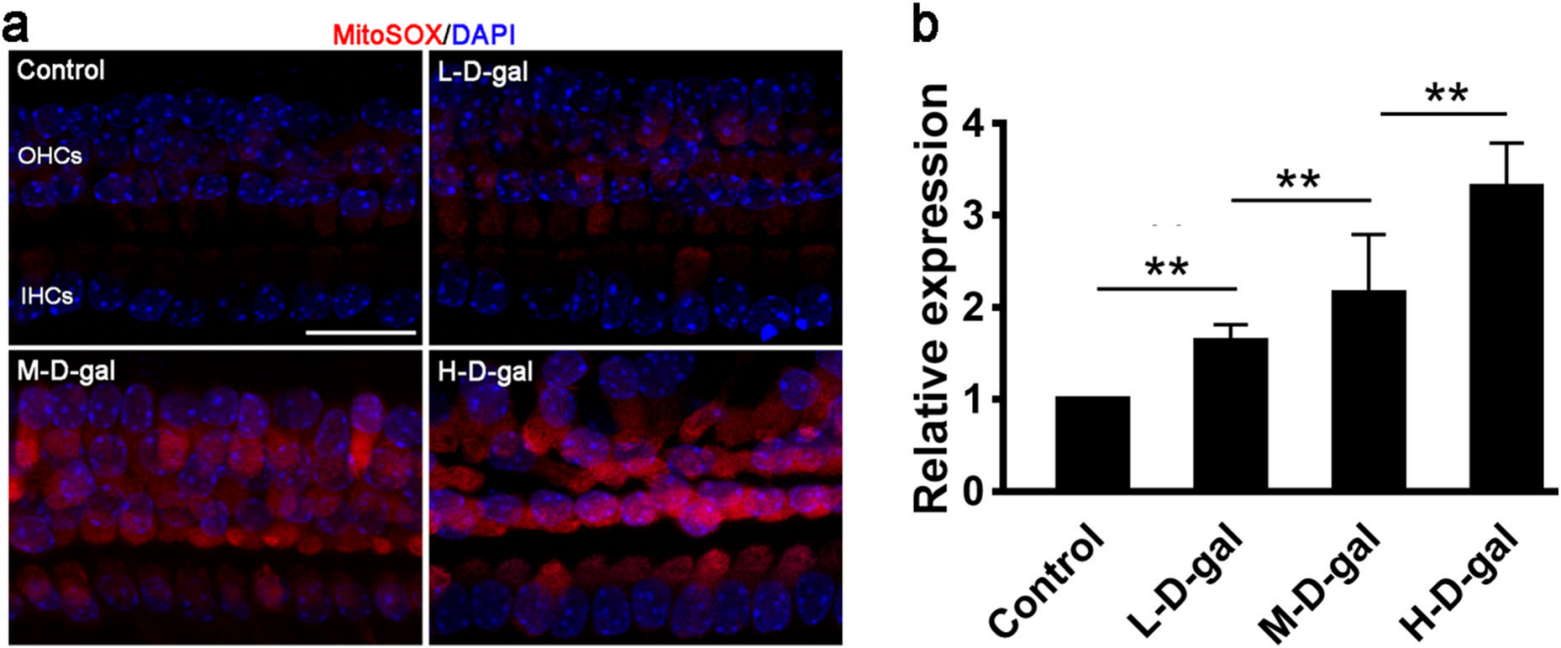
MitoSOX staining and relative levels of reactive oxygen species in the control, low-dose d-gal (L-d-gal), medium-dose d-gal (M-d-gal), and high-dose d-gal (H-d-gal) groups. **a** Representative images of MitoSOX staining (red) in the different groups. **b** Relative MitoSOX red fluorescence intensity in the different groups Data are shown as means ± SD (n = 6 in each group). *P < 0.05, **P < 0.01. Scale bar = 20 μm. DAPI, 4′,6-diamidino-2-phenylindole

The levels of mitochondrial ROS were measured by MitoSOX staining. As shown in Fig. 3a, MitoSOX red staining in the d-gal-treated groups was significantly higher than in the control group. MitoSOX staining was concentration-dependent with the highest number of stained cells in the H-d-gal group. Compared with the control group, relative staining in the L-d-gal, M-d-gal, and H-d-gal groups were found to be increased by 1.64 ± 0.07-fold, 2.33 ± 0.17-fold, and 3.31 ± 0.20-fold, respectively (Fig. 3b). These results indicate that mitochondrial ROS generation was increased by d-gal in the CBM in vitro.

### Mitochondrial CD accumulation in CBMs induced by d-gal

**Fig. 4:**
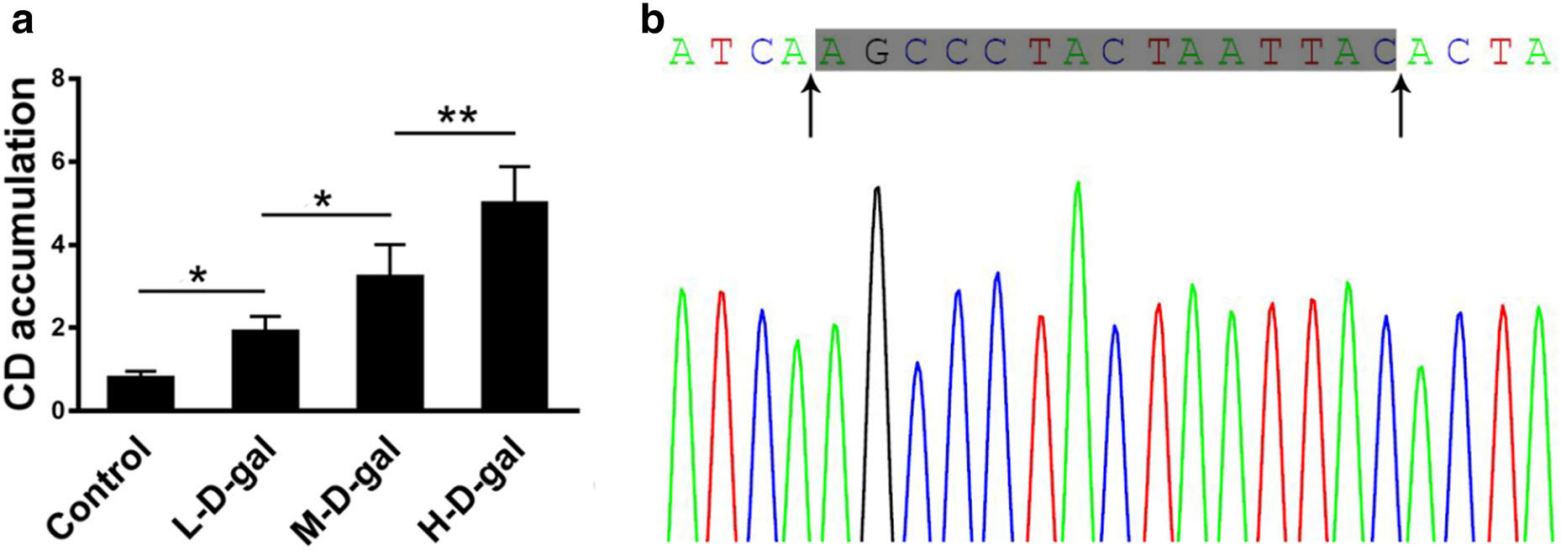
Detection of a mitochondrial CD in the control, low-dose d-gal (L-d-gal), medium-dose d-gal (M-d-gal), and high-dose d-gal (H-d-gal) groups. **a** Quantitative analysis of the accumulation of the mitochondrial CD in the different groups. **b** CD polymerase chain reaction product sequencing. Arrows point to the two putative breakpoint sites. The grey area marks the 15-bp direct repeat sequence. Data are shown as means ± SD (n = 4 in each group). *P < 0.05, **P < 0.01

Mitochondrial DNA mutations were evaluated by detecting a CD. Compared with the control group, the accumulation of the CD in the L-d-gal, M-d-gal, and H-d-gal groups were found to be increased by 1.90 ± 0.15-fold, 3.23 ± 0.32-fold, and 4.99 ± 0.36-fold, respectively (Fig. 4a). The CD PCR product was sequenced and showed a 15-bp direct repeat sequence (Fig. 4b). These results demonstrate that a CD accumulates in the CBM mitochondria following treatment with d-gal in vitro.

### Mitochondrial dysfunction in CBMs induced by d-gal

**Fig. 5:**
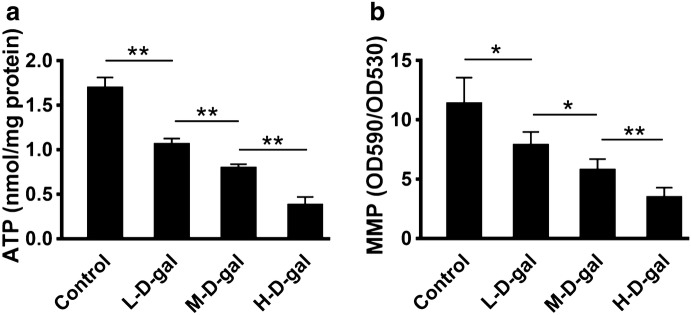
Levels of ATP and the mitochondrial membrane potential (MMP) in the control, low-dose d-gal (L-d-gal), medium-dose d-gal (M-d-gal), and high-dose d-gal (H-d-gal) groups. **a** ATP levels in each group. **b** The MMP in each group. Data are shown as means ± SD (n = 6 in each group). *P < 0.05, **P < 0.01

To assess mitochondrial function in each treatment group, we analyzed the levels of both ATP and MMP. As shown in Fig. 5a, ATP levels in the control, L-d-gal, M-d-gal, and H-d-gal groups were found to be 1.70 ± 0.05, 1.06 ± 0.03,0.787 ± 0.02, and 0.38 ± 0.04 nmol/mg protein, respectively. ATP level in the L-d-gal group was significantly lower than that in the control group (*P* < 0.01). ATP level in the M-d-gal group was significantly lower than that in the L-d-gal group (*P* < 0.01). ATP level in the H-d-gal group was significantly lower than that in the M-d-gal group (*P* < 0.01). MMP levels in the control, L-d-gal, M-d-gal, and H-d-gal groups were 11.39 ± 0.97, 7.88 ± 0.49, 5.78 ± 0.41, and 3.45 ± 0.37, respectively (Fig. 5b). MMP level in the L-d-gal group was lower than that in the control group (*P* < 0.05). MMP level in the M-d-gal group was lower than that in the L-d-gal group (*P* < 0.05). MMP level in the H-d-gal group was lower than that in the M-d-gal group (*P* < 0.01). These results suggest that d-gal causes a concentration-dependent decline of mitochondrial function in the CBM in vitro.

### Hair cell stereocilia bundle damage in CBMs treated with d-gal

**Fig. 6:**
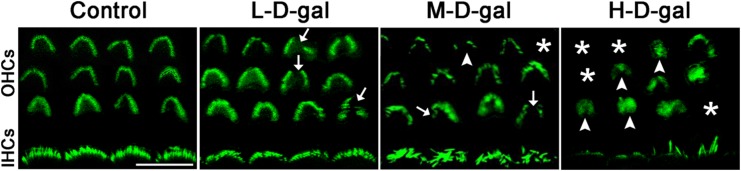
Representative immunofluorescence images of outer hair cells (OHCs) and inner hair cells (IHCs) stereocilia bundles in the control, low-dose d-gal (L-d-gal), medium-dose d-gal (M-d-gal), and high-dose d-gal (H-d-gal) groups. Complete and orderly V-shaped stereocilia bundles are arranged on OHCs, and complete and orderly stereocilia bundles are arranged on IHCs in the control group. Stereocilia breakage (arrows), degeneration (arrowheads), and loss (asterisks) are observed in the D-gal-treated groups. Scale bar = 10 μm

Changes of hair cell stereocilia bundles were assessed by immunofluorescence staining (Fig. 6). In the control group, we observed three rows of the OHC stereocilia bundles that were in an inverted V-shape, and one row of the IHC stereocilia bundles that was complete and orderly. Minor changes involving fusion or degeneration of the OHC stereocilia were observed in the L-d-gal group, but the IHC stereocilia were maintained. In the M-d-gal group, the OHC stereocilia began to decline and the IHC stereocilia showed a disordered arrangement. In the H-d-gal group, the OHC stereocilia V-shaped construction disappeared, and a large quantity of the OHC stereocilia and the IHC stereocilia were lost. These results indicate that increasing concentrations of d-gal causes increasing damage to the hair cell stereocilia bundles in the CBM in vitro.

### Ribbon synapse, IHC, and OHC loss in the CBM induced by d-gal

**Fig. 7:**
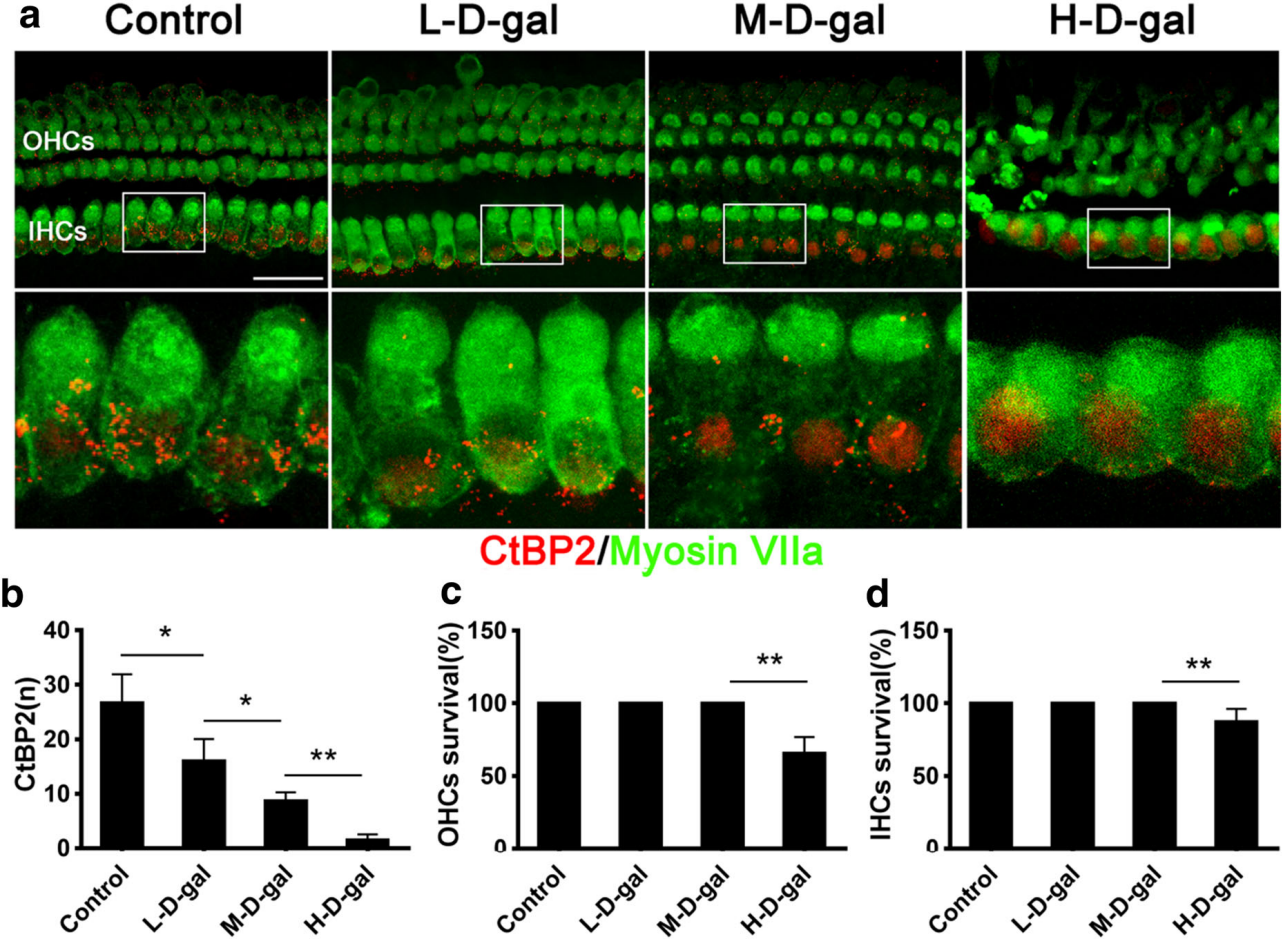
Quantification of ribbon synapses, inner hair cells (IHCs), and outer hair cells (OHCs) in the control, low-dose d-gal (L-d-gal), medium-dose d-gal (M-d-gal), and high-dose d-gal (H-d-gal) groups. **a** Representative immunofluorescence images of ribbon synapses (red), and IHCs and OHCs (green) in each group. **b** The number of ribbon synapses per IHC in each group. **c** OHCs survival in each group. **d** IHCs survival in each group. Data are shown as means ± SD (n = 6 in each group). *P < 0.05, **P < 0.01. Scale bar = 25 μm

We observed quantitative changes of the ribbon synapses, IHCs, and OHCs by immunofluorescence staining in each treatment group (Fig. 7a). As shown in Fig. 7b, the numbers of ribbon synapses in the control, L-d-gal, M-d-gal, and H-d-gal groups were 26.7 ± 2.1, 16.0 ± 1.7, 8.67 ± 0.67, and 1.5 ± 0.43, respectively. The number of ribbon synapses in the L-d-gal group was lower than that in the control group (*P* < 0.05). The number of ribbon synapses in the M-d-gal group was lower than that in the L-d-gal group (*P* < 0.05). The number of ribbon synapses in the H-d-gal group was lower than that in the M-d-gal group (*P* < 0.01). However, a loss of IHCs and OHCs was only observed in the H-d-gal group. As shown in Fig. 7b, c, the survival percentages of OHCs and IHCs were 65.8 ± 4.5 and 87.7 ± 3.5%, respectively. The loss of OHCs and IHCs in the H-d-gal group was significantly different from the other groups. These findings indicate that d-gal damages ribbon synapses and hair cells in the CBM in vitro.

### Caspase 3-dependent apoptosis in the CBM induced by d-gal

**Fig. 8:**
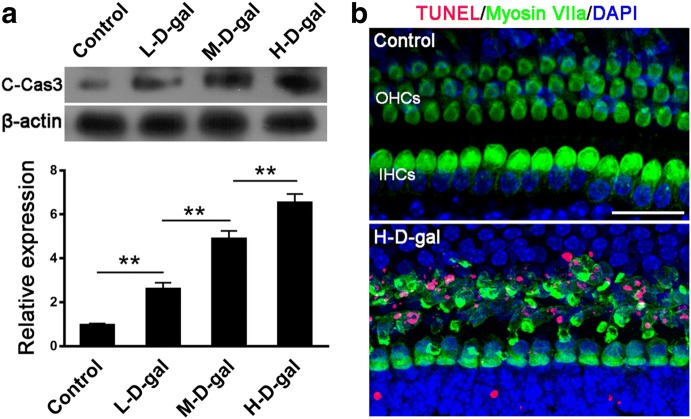
Caspase 3-dependent apoptosis in the control, low-dose d-gal (L-d-gal), medium-dose d-gal (M-d-gal), and high-dose d-gal (H-d-gal) groups. **a** Protein expression of cleaved-caspase-3 (C-Cas3) in each group. **b** TUNEL-positive cells (red) in the OHCs (green) area of the H-d-gal group. Data are shown as means ± SD (n = 4 in each group). *P < 0.05, **P < 0.01. Scale bar = 25 μm

The occurrence of apoptosis was confirmed by western blots of C-Cas3 (Fig. 8a) and TUNEL staining (Fig. 8b). Compared with the control group, the levels of C-Cas3 protein in the L-d-gal, M-d-gal, and H-d-gal groups were found to be increased by 2.61 ± 0.17-fold, 4.89 ± 0.15-fold, and 6.54 ± 0.22-fold, respectively. The level of C-Cas3 protein in the L-d-gal group was significantly higher than that in the control group (*P* < 0.01). The level of C-Cas3 protein in the M-d-gal group was significantly higher than that in the L-d-gal group (*P* < 0.01). The level of C-Cas3 protein in the H-d-gal group was significantly higher than that in the M-d-gal d-gal d-gal group (*P* < 0.01). Apoptotic CBM cells were detected by TUNEL staining. As shown in Fig. 8b, the TUNEL-positive cells were only observed in the OHC area of the H-d-gal group. There were no TUNEL-positive cells in the hair cell area of the CBM in the control, L-d-gal, or M-d-gal groups. These findings indicate that d-gal activates caspase 3-dependent apoptosis in the CBM in vitro.

## Discussion

Hearing relies on the mechanical-electrochemical conversion activity of hair cells (Taylor et al. 2015) and the electrochemical transmission of the IHC ribbon synapses (Sergeyenko et al. 2013) on the CBM. The loss or degeneration of hair cells and the ribbon synapses are the most important reasons for presbycusis (Kidd and Bao 2012; Xie and Manis 2017). Because of the inaccessibility of human cochlear tissue in live patients, the study of presbycusis is limited. Thus, animal models of aging have been widely used in research.

d-gal is a reducing sugar. When its concentration reaches a certain level, metabolic disorders occur, contributing to the generation of ROS, ultimately resulting in aging (Saleh et al. 2019). d-gal d-gal has been used to successfully study aging in many organs, with pathological changes that are similar to the process of natural aging in mice (Cui et al. 2006; Du et al. 2012, 2019b; Liu et al. 2010; Zhang et al. 2010).

In the present study, we demonstrated that d-gal can induce CBM senescence in vitro. Previous studies indicated that the SA-β-Gal has been associated with cellular aging and considered to be a specific senescence biomarker (Bodnar et al. 1998; Zhao et al. 2016). Specifically, staining images for SA-β-Gal showed that d-gal induced positive staining (from light to dark blue color) of cells on the CBM compared with the control group. These characteristic staining features are consistent with the cell aging process.

Aging is related to an increase of mitochondrial ROS production (Hiona and Leeuwenburgh 2008). ROS are produced by electron transport in normal metabolic activities and participate in the signal transduction process of the mitochondria (Kamsler et al. 2001). High levels of ROS can inflict oxidative damage to cellular components, induce oxidation of the mitochondrial DNA and proteins, and result in cell aging (Kujoth et al. 2005; Yu and Bennett 2016). In this study, MitoSOX staining demonstrated that increasing concentrations of d-gal caused increasing ROS and damage to the CBM.

For the mitochondria, the cumulative burden of ROS production in cells can cause mutations in the mitochondrial DNA (Hiona and Leeuwenburgh 2008). Among these mutations, the accumulation of CDs exhibits the strongest correlation with aging and is considered to be an ideal biomarker of aging (Zhong et al. 2011). Previous research showed that the accumulation of CDs in the human inner ear tissue had a close association with the development of presbycusis. One study found that a 4977-bp deletion was frequently found and increased in aged human cochlear tissues (Markaryan et al. 2009). Interestingly, the 3860-bp deletion of the mitochondrial DNA in mice is closely located to the 4977-bp deletion in humans, and can also be found in various aged tissues. Furthermore, its occurrence increases in the auditory nervous system, in the liver, and in the brain with aging (Du et al. 2019a; Zhang et al. 2009, 2013). In our study, the 3860-bp CD increased gradually in the d-gal-treated CBM compared to increased levels observed in the control CBM. Therefore, these findings suggest that d-gal increased mitochondrial ROS production and CD accumulation.

Mitochondria are the cellular power sources, for they produce ATP. Mitochondrial oxidative damage and CD accumulation may lead to energy deficiency and mitochondrial dysfunction (Du et al. 2015, 2019b). Specifically, the oxidation-damaged mitochondria produce less ATP and have a reduced MMP. In our study, the levels of both ATP and MMP in each d-gal group were significantly decreased as compared to that in the control group. According to the mitochondrial aging theory, CD accumulation and mitochondrial dysfunction may lead to aging and age-related diseases (Elfawy and Das 2019; Nicklas et al. 2004; Zhong et al. 2011).

The results of our study also provide a quantitative description of the IHC ribbon synapses and hair cell loss or degeneration induced by d-gal. Hearing is dependent on the normal function of the cochlear hair cells and the IHC ribbon synapses. The transport, aggregation, and quantum release of synaptic vesicles are key to maintaining normal synapse functions (Matthews and Fuchs 2010). During normal physiological function of the IHC ribbon synapse, large amounts of ATP, produced by IHC mitochondria, are consumed. Therefore, ATP levels play a vital role in maintaining synaptic function (Li et al. 2004; Verstreken et al. 2005).

In the present study, exposure to d-gal significantly decreased the number of the IHC ribbon synapses. The concomitant decrease in ATP levels provides insufficient energy to transport ribbon synaptic vesicles in the IHCs, decreasing the number and function of the IHC ribbon synapses. The impairment of the IHC ribbon synapses caused by aging reduces nerve impulse signals to the central auditory nervous system, leading to a loss of hearing. Related studies showed that an age-related synapses loss could be detected a few weeks before changes of the auditory threshold measured by the auditory brainstem response in rats (Cai et al. 2018). This suggests that the ribbon synapses connecting IHCs and spiral ganglion neurons are a significant marker of early presbycusis.

We also found that the degeneration of stereocilia bundles was concentration-dependent when exposed to d-gal. Hair cell stereocilia bundles are essential to the perception of sound and motion in the auditory transmission system (Liu et al. 2019). Previous studies have reported that diverse forms of cochlear hair cell stereocilia bundle degeneration occurred as early as 2 weeks of age in the C57BL/6J-, A/J-, NOD/LtJ-, and DBA/2J- mice. This suggests that cochlear hair cell stereocilia degeneration may be one of the causes of early age-related hearing loss in these mice strains (Li and Hultcrantz 1994; Liu et al. 2019). Therefore, we hypothesize that the degeneration or loss of hair cell stereocilia may contribute to an early loss of hearing.

In the present study, we observed a massive loss of OHCs and a small loss of IHCs in the CBM of the H-d-gal group. In the process of sound transmission, IHCs convert sound-induced mechanical signals into electrophysiological signals and transmit them to the central auditory system. OHCs with special mechanical feeling can amplify the sensitivity of hearing by basilar membrane vibration. Interestingly, one study reported that some strains of mice showed priority and massive loss of OHCs compared to a subsequent small loss of IHCs in the middle and in the later stages of life (Spongr et al. 1997).

In our study, we confirmed that the loss of OHCs in the CBM of the H-d-gal group was largely because of the activation of apoptosis. Other evidence shows that apoptosis plays an important role in aging and aging-related degenerative diseases (Kujoth et al. 2005). Studies of apoptosis demonstrate that the two major induction pathways are the mitochondrial apoptotic pathway and the death receptor pathway. The mitochondrial apoptotic pathway plays an important role in mammals. Mitochondrial dysfunction leads to an irreversible opening of the permeability transition pore and increases mitochondrial outer membrane permeability in the mitochondrial pathway of apoptosis (Warnsmann et al. 2018; Zamzami et al. 1997). Subsequently, the MMP is decreased and the mitochondria release apoptosis-inducing factor and cytochrome c into the cytosol. This activates (i.e., cleaves) caspase-3 and induces apoptosis (Du et al. 2019b; Kujoth et al. 2005). Our findings suggest that the mitochondrial pathway of apoptosis leads to aging and may be involved in the loss of OHCs in the CBM induced by d-gal.

Overall, we established a new and a reliable model of auditory aging in cultured CBM using d-gal. Importantly, we were able to imitate different stages of presbycusis in the CBM in vitro. This provides a useful in vitro model to investigate the mechanisms of presbycusis.
